# Transcriptome Analyses Reveal Effects of Vitamin C-Treated Donor Cells on Cloned Bovine Embryo Development

**DOI:** 10.3390/ijms20112628

**Published:** 2019-05-28

**Authors:** Lei Zhang, Yan Zhang, Zhuo Han, Jingshuai Fang, Huanhuan Chen, Zekun Guo

**Affiliations:** 1 College of Veterinary Medicine, Northwest A&F University, Yangling, Shaanxi 712100, China; zlei@nwafu.edu.cn (L.Z.); ryan.yan.chang@gmail.com (Y.Z.); hz1991@nwafu.edu.cn (Z.H.); jsfang911@126.com (J.F.); chh@nwafu.edu.cn (H.C.); 2 Key Laboratory of Animal Biotechnology, Ministry of Agriculture, Northwest A&F University, Yangling, Shaanxi 712100, China

**Keywords:** transcriptomics, nuclear transfer, reprogramming, L-ascorbic acid, *cattle*

## Abstract

Somatic cell nuclear transfer (SCNT) is a very powerful technique used to produce genetically identical or modified animals. However, the cloning efficiency in mammals remains low. In this study, we aimed to explore the effects of vitamin C (Vc)-treated donor cells on cloned embryos. As a result, Vc treatment relaxed the chromatin of donor cells and improved cloned embryo development. RNA sequencing was adopted to investigate the changes in the transcriptional profiles in early embryos. We found that Vc treatment increased the expression of genes involved in the cell–substrate adherens junction. Gene ontology (GO) analysis revealed that Vc treatment facilitated the activation of autophagy, which was deficient in cloned two-cell embryos. Rapamycin, an effective autophagy activator, increased the formation of cloned blastocysts (36.0% vs. 25.6%, *p* < 0.05). Abnormal expression of some coding genes and long non-coding RNAs in cloned embryos was restored by Vc treatment, including the zinc-finger protein 641 (ZNF641)*. ZNF641* compensation by means of mRNA microinjection improved the developmental potential of cloned embryos. Moreover, Vc treatment rescued some deficient RNA-editing sites in cloned two-cell embryos. Collectively, Vc-treated donor cells improved the development of the cloned embryo by affecting embryonic transcription. This study provided useful resources for future work to promote the reprogramming process in SCNT embryos.

## 1. Introduction

Animal cloning has been successful since 1997 when “Dolly the *sheep*” was first created by nuclear transfer using differentiated somatic cells (SCNT) [1]. *Cattle* were cloned from adult cells in 1998 [2]. However, cloning efficiency remains low, represented by low birth rates and several health problems in the offspring, especially for large livestock [3,4]. Determining the underlying mechanism of genetic reprogramming to improve the nuclear transfer (NT) procedures and optimize the outcomes of SCNT has proven to be difficult. However, in fertilized embryos, a series of reprogramming events occur efficiently.

Incomplete nuclear reprogramming is the most serious problem in cloned embryos [5,6]. During the production of SCNT embryos, the epigenetic memory of donor somatic cells must be erased to ensure appropriated embryonic access to the genome. However, the donor cells are highly differentiated and difficult to radically reprogram into a pluripotent state through epigenetic modifications [7]. Especially, aberrant DNA hypermethylation was observed in the cloned embryos [8,9]. Drugs that modify and remold the epigenome have been widely studied in the context of SCNT.

Vitamin C (Vc) promotes epigenetic reprogramming [10,11,12] and can induce ten-eleven-translocation (TET)-mediated DNA demethylation in both embryonic stem cells [13,14] and somatic cells [15,16,17]. Indeed, Vc has been demonstrated to promote somatic cell reprogramming during the generation of induced pluripotent stem cells [18]. The results of our previous research [19] revealed that Vc treatment can reduce the methylation level of donor nuclei and improve cloned embryo development in *cattle*. The mechanisms of Vc to promote cloned embryo development need to be further investigated. Donor nuclei with modified epigenetics by Vc treatment probably change the gene expression in cloned embryos. The potential role of Vc in transcriptional activation has been proved in human skin fibroblasts. Vc promoted the expression of genes related to DNA replication and repair and to the G2/M phase of the cell cycle [20]. Besides, type I and Type III procollagen messenger RNA levels were raised by Vc treatment [21,22]. To our knowledge, the influences of Vc treatment on the gene expression in cloned embryos have not been studied.

Several genes and processes that are involved in normal embryo development are the focus of this investigation for the cloned embryos produced by Vc-treated donor cells. These genes and processes include autophagy, *ZNF641*, lncRNAs and RNA-editing. Autophagy is involved in the degradation of maternal proteins after fertilization [23]. Autophagic degradation is essential for preimplantation embryo development [24,25]. *ZNF641* can activate the transcriptional activities of serum response element (SRE) and activator protein 1 (AP-1), most likely by MAPK-mediated signaling pathways [26]. LncRNAs participate in many developmental processes, such as genomic imprinting, dosage compensation, cell differentiation, and organogenesis [27]. Adenosine (A) to inosine (I) RNA-editing is mediated by ADAR enzymes (adenosine deaminase acting on RNA) in double-stranded RNA molecules. During early human embryogenesis, dynamic changes in RNA-editing were revealed by single-cell analysis [28]. In mouse oocytes and preimplantation embryos, some edited microRNA precursors could be selectively eliminated [29].

In this study, we first investigated the influence of Vc treatment on the extent of chromatin compaction in donor cells. Then, the effects of Vc treatment on the gene expression in cloned embryos were examined using RNA sequencing. Genes and processes rescued by Vc treatment in cloned embryos were analyzed and their biological functions were studied in the context of SCNT.

## 2. Results

### 2.1. Global View of Mapped Reads from RNA Sequencing

The filtered data from RNA sequencing were assessed, including read numbers, saturation and the distribution of mapped reads across whole-genome regions. The mapped reads covered most of the genes that were identified (Figure S1). The “gene” and “coding” regions were mainly enriched by the mapped reads (Figure S2).

### 2.2. Vc Treatment Relaxed Chromatin of Donor Cells and Promoted the Cloned Embryo Development

**Figure 1:**
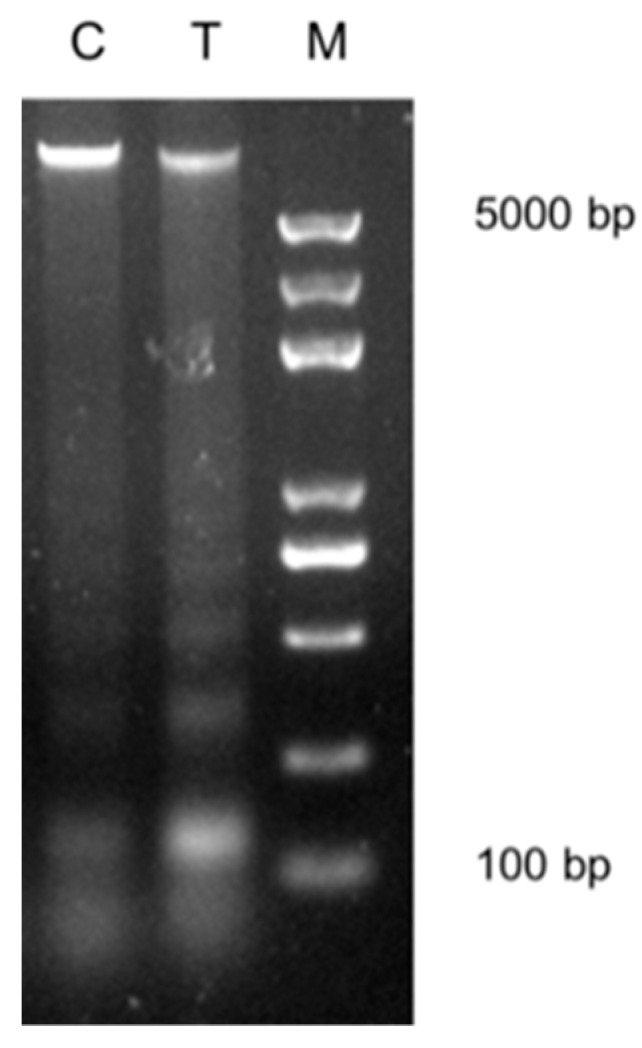
Effects of vitamin C (Vc) treatment on the higher-order chromatin structure in somatic cell nuclear transfer (SCNT) donor cells. Donor nuclei were digested with MNase under the same conditions for both the control cells (C) and the Vc-treated cells (T). DNA marker (M) was used.

In our previous study, Vc was found to promote cloned embryo development [19]. In this study, another donor cell line for SCNT was treated with Vc under the same conditions. As a result, Vc also improved the development of the cloned embryo (Table 1). The cleavage of cloned embryos was slower than IVF embryos on day 1 (51.8% vs 68.5%, *p* < 0.05) and was accelerated using Vc-treated donor cells (65.0% vs 51.8%). The blastocyst rate was higher in the Vc-treated group than in the control group (42.2% vs. 27.6%, *p* < 0.05), attaining a rate comparable to that of IVF embryos. We had found that Vc treatment decreased DNA methylation in donor cells; therefore, we speculated that Vc treatment might relax chromatin of donor cells. The MNase digestion assay was adopted to examine the extent of chromatin compaction. We found the donor nuclei were more susceptible to MNase digestion and were easily digested into smaller nucleosome fragments after Vc treatment (Figure 1).

### 2.3. Vc Treatment Raised the Expression of Genes Coding for Cell–Substrate Adherens Junction in the Cloned Embryos

RNA sequencing was used to examine the effect of Vc treatment on gene expression in cloned embryos. The statistical parameters for differentially expressed genes (DEGs) were: *p* < 0.01; FPKM > 1 in at least one group; fold change (FC) > 2. We focused on the genes that were upregulated in the cloned embryos produced by Vc-treated donor cells.

**Figure 2:**
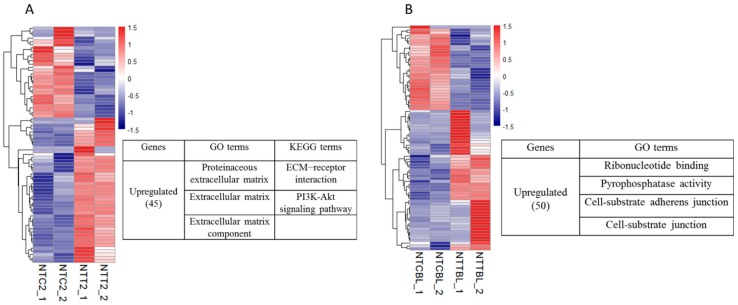
Effects of Vc treatment on the gene expression in cloned embryos. A heatmap illustration showing the genes affected by Vc treatment in the cloned embryos. Differentially expressed genes (DEGs) were analyzed under the conditions of *p* < 0.01, fold change (FC) > 2, and fragments per kilobase of exon per million reads mapped (FPKM) > 1 in at least one group. Genes that were upregulated by Vc treatment were enriched by gene ontology (GO) and KEGG analysis. (**A**) Gene expression changes in the two-cell stage. (**B**) Gene expression changes in the blastocyst stage. NTC2/NTCBL, cloned two-cell embryos or blastocysts produced by the control donor cells; NTT2/NTTBL, cloned two-cell embryos or blastocysts produced by the Vc-treated donor cells.

First, we analyzed the effect of Vc treatment on gene expression in the cloned two-cell embryos. Among the 73 changed genes, 45 were more highly expressed in the NTT two-cell embryos (Vc-treated) than in the NTC two-cell embryos (control). Gene ontology (GO) analysis revealed that these genes were significantly enriched in categories specific to “extracellular matrix (ECM)”. For Kyoto Encyclopedia of Genes and Genomes (KEGG) analysis, “ECM-receptor interaction” and “PI3K-Akt signaling pathway” were enriched (Figure 2A). Then, we analyzed the effect of Vc treatment on gene expression in the cloned blastocysts. Among the 80 changed genes, 50 were upregulated in the NTT blastocysts compared to the NTC blastocysts. The GO terms, “ribonucleotide binding”, “pyrophosphatase activity”, “cell-substrate adherens junction” and “cell-substrate junction”, were enriched (Figure 2B). In summary, Vc treatment increased the expression of genes specific to the extracellular matrix and cell–substrate adherens junction in the cloned embryos.

### 2.4. Vc Treatment Improved Autophagy Activation in the Cloned Two-Cell Embryos

In this study, we analyzed the effect of Vc treatment on the activation of autophagy in cloned two-cell embryos. As a result, the GO term “autophagy” was significantly enriched for genes that were upregulated in the IVF two-cell embryos compared to MII oocytes (FC > 3, FPKM > 1 in the IVF two-cell embryos). However, “autophagy” was included in the enriched GO terms for genes that were downregulated in the NTC two-cell embryos compared to the IVF two-cell embryos. Thus, we speculated that the activation of autophagy was insufficient in the cloned two-cell embryos. Interestingly, there was no difference in autophagy activation between the NTT and IVF two-cell embryos. This suggested that the deficient autophagy activation in the cloned two-cell embryos could be restored by Vc treatment. Then, we identified genes that were associated with autophagic activation and were upregulated in the NTT two-cell embryos compared to the NTC two-cell embryos. GO analysis recognized five genes related to the regulation of autophagy, including *MTCL1, DCN, KDR, SH3BP4* and *TPCN2*. There were another three genes related to autophagosome assembly, including *ATG9B, EPG5* and *ATG4C*.

To further examine the role of autophagy on the cloned embryo development, rapamycin, an effective autophagy activator was used to treat the cloned embryos. After the first cleavage of the constructed zygotes, 10 nM rapamycin was supplemented into the embryo culture medium for 6 h. As a result, blastocyst formation was improved (36.0% vs. 25.6%; *p <* 0.05; Table 2).

### 2.5. Vc Treatment Restored the Expression of Some Coding Genes in the Cloned Embryos

Gene expression was analyzed under the conditions of *p <* 0.01, FC > 2, and FPKM > 1 in at least one group. The genes were considered as being completely restored by Vc treatment if they met the following criteria: The abundance of a gene was different between NTC and both IVF and NTT, and there was no difference in abundance between IVF and NTT.

**Figure 3:**
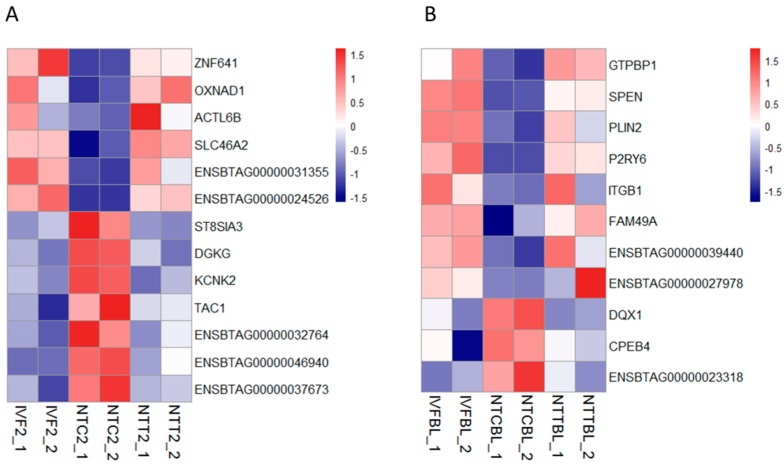
Restored gene expression in cloned embryos by Vc treatment. Heatmap illustration showing the genes that were restored by Vc treatment in cloned embryos compared to their in vitro fertilization (IVF) counterparts. Differentially expressed genes (DEGs) were analyzed under the conditions of *p* < 0.01, FC > 2, and FPKM > 1 in at least one group. (**A**) Restored gene expression in the two-cell stage. (**B**) Restored gene expression in the blastocyst stage. IVF2/IVFBL, in vitro fertilized two-cell embryos or blastocysts.

First, genes that were rescued in the cloned two-cell embryos were determined. As a result, 13 genes were completely rescued by Vc treatment (Figure 3A). Among them, six genes were aberrantly decreased in the NTC two-cell embryos compared to the IVF two-cell embryos. The expression of *ZNF641* and *OXNAD1* was validated by RT-qPCR (Figure S3). Then, genes that were rescued in the cloned blastocysts were determined. As a result, 11 genes were completely rescued by Vc treatment (Figure 3B). Among them, eight genes were abnormally decreased in the NTC blastocysts compared to those in the IVF blastocysts. The expression of *GTP binding protein 1* (*GTPBP1*) was validated by RT-qPCR (Figure S3).

**Figure 4:**
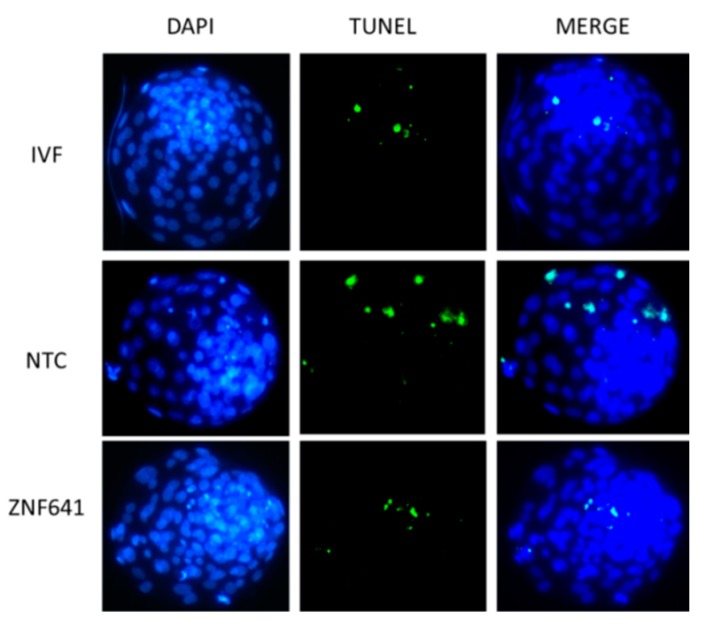
Effects of ZNF641 overexpression on the apoptosis in cloned blastocysts. Apoptosis in blastocysts was reported by TUNEL staining (green). DNA was stained by DAPI (blue). The total and apoptotic cell numbers in each individual blastocyst are indicated in Table 3. IVF, in vitro fertilized embryos; NTC, cloned embryos; ZNF641, the cloned embryos injected with ZNF641 mRNA.

**Figure 5:**
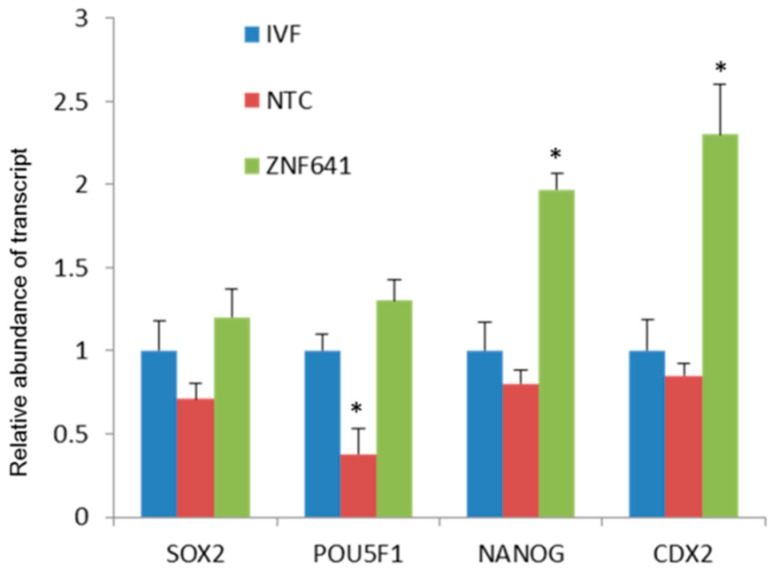
Effects of ZNF641 overexpression on the pluripotent genes in cloned blastocysts. The abundance of a gene transcript was set as “1” in the IVF blastocysts and the relative expressions were calculated in the cloned embryos. In the same cluster, an asterisk superscript indicates the difference relative to the IVF counterpart (*p* < 0.05).

*ZNF641* was not activated in the cloned two-cell embryos and was rescued by Vc treatment. We speculated that the restored *ZNF641* expression by Vc treatment might be partially responsible for the improved development of cloned embryos. To examine the effects of *ZNF641* compensation on the development of the cloned embryo, *ZNF641* mRNA was generated and injected into constructed one-cell embryos. As a result, it was not definitive that the embryonic development was promoted by *ZNF641* overexpression (30.8% vs. 24.3%, *p* > 0.05). However, the quality of derived blastocysts was improved, indicated by increased total cell numbers, decreased apoptosis (Figure 4, Table 3), and raised expression of pluripotent genes (Figure 5).

### 2.6. Vc Treatment Improved Long Non-Coding RNA Expression in the Cloned Embryos

To further investigate the effects of Vc treatment on long non-coding RNA (lncRNA) expression, lncRNAs that were abnormally expressed in cloned embryos but restored by Vc treatment were identified. Under conditions of *q* < 0.05, FC > 2, and FPKM > 1 in at least one group, lncRNAs were considered to be restored by Vc treatment when they met the following criteria: The abundance was different between NTC and both IVF and NTT embryos, and there was no difference between the abundance in the IVF and NTT embryos.

**Figure 6:**
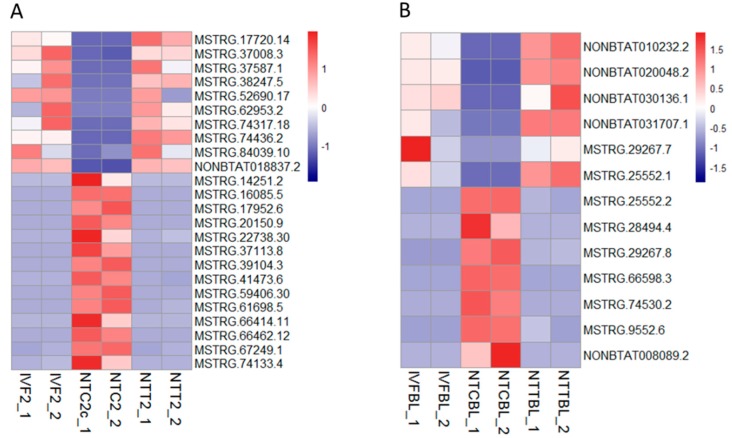
Restored lncRNA expression by Vc treatment in the cloned embryos. Heatmap illustration showing the lncRNAs that were restored by Vc treatment in the cloned embryos compared to their IVF counterparts. Differentially expressed genes (DEGs) were analyzed under the conditions of *q* < 0.05, FC > 2, and FPKM > 1 in at least one of the three groups. (**A**) Restored lncRNAs in the two-cell stage. (**B**) Restored lncRNAs in the blastocyst stage.

As a result, a series of lncRNAs were restored by Vc treatment in the cloned two-cell embryos and blastocysts (Figure 6). There were 24 lncRNAs included in the two-cell stage—10 that ectopically decreased and 14 that ectopically increased in the cloned embryos. In the blastocyst stage, there were 13 lncRNAs—six that were abnormally reduced and seven that were abnormally elevated in the cloned blastocysts. Transcription amounts of all of the above lncRNAs were completely rescued by Vc treatment.

### 2.7. Vc Treatment Restored Some Deficient RNA-Editing Sites in the Cloned Two-Cell Embryos

We analyzed the effect of Vc treatment on the possible RNA-editing defects in the cloned embryos. The deficient RNA-editing sites were judged as improved by Vc treatment after meeting the following criteria: The variation frequency was lower in NTC two-cell embryos than in IVF two-cell embryos (FC > 2, *p* < 0.05 and FDR < 0.2), and it was increased in NTT two-cell embryos compared to NTC two-cell embryos (FC > 2, *p* < 0.2). As a result, Vc treatment improved the RNA-editing of 14 sites in the cloned two-cell embryos, including one synonymous variation and two 3′-untranslated coding regions (UTR) sites (Table 4).

## 3. Materials and Methods

All chemicals and reagents were purchased from Sigma-Aldrich Inc. (St. Louis, MO, USA) unless indicated otherwise. Experimental procedures were approved by the Animal Care Commission of the College of Veterinary Medicine, Northwest A and F University, P. R. China (code: 160822, date: 27/09/2016).

### 3.1. Oocyte Collection and Embryo Culture

Bovine MII oocytes were harvested by in vitro maturation of germinal vesicle oocytes collected from ovaries obtained from a slaughterhouse. Fertilized or constructed embryos were cultured in G1.5/G2.5, a series of culture media (Vitrolife AB, Gothenburg, Sweden). Detailed procedures can be found in our previous study [30].

### 3.2. Somatic Cell Nuclear Transfer and In Vitro Fertilization

Somatic cell nuclear transfer (SCNT) was conducted based on our previous report [19]. Briefly, a glass pipette was used to aspirate the first polar body and the adjacent cytoplasm of MII oocytes. Then, a single donor cell was injected into the perivitelline space of an enucleated oocyte. Cell–oocyte fusion was accomplished with the use of electrical pulses on their contact surface. Reconstructed embryos were activated by ionomycin, combined with dimethylaminopyridine (DMAP). In vitro fertilization (IVF) procedures were also similar to that of the previous study [19]. Briefly, frozen spermatozoa were thawed and achieved capacitation in vitro. Maturated cumulus–oocyte complexes (COCs) were mixed with a sperm suspension (2 × 10^6^ spermatozoa/mL).

### 3.3. Vc Treatment of Donor Cells

The donor fibroblasts were treated with Vc for three days, with fresh culture medium and newly supplemented Vc used each day (A8960; Sigma-Aldrich). Based on our previous study [19], 0.15 mM Vc was selected as an adequate concentration to treat bovine donor fibroblasts. When nearly confluent, the Vc-treated fibroblasts were digested and used as donor cells for SCNT.

### 3.4. Collection of Samples for RNA Sequencing

Oocytes or embryos were fully washed in RNase free polyvinyl alcohol-phosphate-buffered saline (PVA-PBS). Ten oocytes or embryos for each sample were collected in lysis buffer containing Triton X-100 and RNase-out in water. The samples were then immediately preserved in an ultralow-temperature freezer.

### 3.5. Library Preparation and Sequencing

Embryo and oocyte samples were amplified and reverse transcribed using the SMART-Seq^®^ v4 Ultra^®^ Low Input RNA Kit (634888, TaKaRa, Tokyo, Japan) to derive enough cDNA. cDNA libraries were then constructed using standard Illumina protocols, containing fragmentation, terminal repair, 3′ end plus A, ligation linker, enrichment and other steps. RNA sequencing was conducted on the successfully amplified and constructed libraries using the next generation sequencing (NGS) platform of Illumina HiSeq2500.

### 3.6. Preprocessing, Mapping, and Gene Expression Analysis

For each sample, the raw reads from RNA sequencing were filtered for adapter sequences. The reads were then filtered from the 3′ end with a quality cutoff of 20. Reads less than 25 bases were discarded. Ribosomal RNA reads belonging to *cattle* species were also removed. Finally, the filtered reads were mapped to the bovine reference genome (UMD 3.1) using Hisat2 v 2.0.4 with its spliced mapping algorithm. Only uniquely mapped reads were used to calculate the number of reads falling into each gene using Stringtie v 1.3.0. Then, the fragments per kilobase of exon per million reads mapped (FPKM) mapped reads were calculated with Perl, after using the trimmed mean of M-values normalization method. The differentially expressed genes between samples were analyzed by edgeR (p-value) and adjusted by multiple hypothesis testing (q-value). The enrichment of differentially expressed genes (DEGs) was analyzed by DAVID Bioinformatics Resources 6.8 (https://david.ncifcrf.gov/). Graphs were produced by functional packages in R v. 3.5.0 (https://www.r-project.org/).

### 3.7. Micrococcal Nuclease Digestion

The control and Vc-treated cells were collected and washed with PBS. For micrococcal nuclease (MNase) digestion, the cells were resuspended in 0.5 mL cold buffer A and incubated for 15 min on ice. The nuclei were then collected by centrifugation and resuspended by MNase dilution buffer. Equal amounts of MNase (1 U) were added to the control and Vc-treated chromatin suspensions, and the chromatin was digested for 10 min at 37 °C. Finally, the reaction was terminated by adding an equal volume of 5 M ethylenediaminetetraacetic acid solution. The above samples were again digested with RNase A for 1 h at 37 °C and with proteinase K for 2 h at 65 °C. Equal amounts of DNA from each sample were then loaded onto 1.5% agarose gels for analysis.

### 3.8. Sample Collection and Quantitative Real-Time PCR

The oocytes and embryos were washed three times with RNase-free PBS-PVA and dissolved in lysis buffer. Without RNA extraction, cDNA for each sample was directly generated using a PrimeScript^TM^ RT reagent Kit (RR047, TaKaRa). The enzyme used for the quantitative PCR reaction was SYBR Premix ExTaq^TM^ II (RR820, TaKaRa), and the instrument we used was a Step One Plus^TM^ Real-Time PCR System (Applied Biosystems, Foster City, CA, USA). The reaction started at 95 °C for 50 s, followed by 40 cycles of 95 °C for 5 s and 60 °C for 30 s. The specificity of primers was observed by the melting curve. The relative quantitative PCR method (2^−ΔΔCt^) was used and *H2A* was selected as the internal reference gene. Primers for amplification were designed and evaluated by NCBI (Table S1).

### 3.9. In Vitro Transcription and mRNA Injection

A bovine *ZNF641* open reading frame was amplified by PCR with a high-fidelity enzyme from the cDNA library derived from skin. A T7 promoter sequence (5′–TAATACGACTCACTATAGG–3′) was included upstream of the forward primer. Fidelity of the amplified T7-*ZNF641* sequences was ensured by DNA sequencing. mRNA was synthesized from the correct PCR products by in vitro transcription using an mMESSAGE mMACHINE^®^ T7 Ultra Kit (AM1345M, Life Technologies, Carlsbad, CA, USA) following the manufacturer’s instructions.

Microinjection of *ZNF641* mRNA was performed at 6 to 7 h post-activation of the constructed embryos. SCNT embryos were injected with about 10 pl of water (control), or 200 ng/μL *ZNF641* mRNA using a micromanipulator.

### 3.10. Apoptosis Assay in Blastocysts

Apoptosis in blastocysts was examined using the TUNEL system (Promega, Madison, WI, USA) based on the procedures described in a previous investigation [31]. Briefly, embryos were fixed with 4% paraformaldehyde and permeated by Triton X-100. Then, embryos were transferred into E-buffer for 5 to 8 min. The following procedures were all carried out in the dark to prevent fluorescence bleaching. Embryos were incubated for 1 h at 37 °C in the apoptosis staining medium. The reaction was terminated with 2× saline sodium citrate. DAPI was used to show the nuclei. Finally, the samples were observed under the fluorescence microscope.

### 3.11. Analysis of RNA-Editing Sites

RNA sequencing data were further analyzed using TopHat and Samtools (mpileup). Single nucleotide variant (SNV) data were derived by Perl script and ANNOVAR. The bovine assembly UMD 3.1 was used as the reference genome. We used only the sequencing bases with a base quality > 20. First, A–G base variations were selected from the SNV data table and filtered with sequencing depth (DP) > 10 and genotype quality (GQ) > 20. Then, variation frequencies (ALT/DP) in both IVF and cloned two-cell embryos were calculated. The minimum variation frequencies in both two IVF replicates were set at 0.1. Variation sites were filtered by known single nucleotide polymorphisms (SNPs) collected from BovineMine (http://bovinegenome.org/bovinemine/begin.do) [32]. Differences in the variation frequencies between IVF and cloned embryos were tested by ANOVA and Benjamini–Hochberg (BH) adjustment.

### 3.12. Statistical Analysis of Data

Each experiment was repeated at least three times. Data are indicated as the mean ± standard error. SPSS 20.0 software (SPSS Inc., Chicago, IL, USA) was used to judge differences among groups by selecting one-way ANOVA and LSD tests. For RT-qPCR analyses, a nonparametric Mann–Whitney U test was adopted. Differences are considered significant when *p* < 0.05.

## 4. Discussion

Vitamin C (Vc) has been widely studied in several reprogramming processes, such as induced pluripotent stem cell generation [18], embryo development [33], and cancer treatment [34]. We demonstrated that Vc-treated donor cells promoted the cleavage of cloned bovine embryos and blastocyst formation in this investigation. In human embryos, earlier cleavage could predict better development to blastocyst and implantation [35]. According to our previous study [19], qualities of cloned blastocysts were improved by Vc treatment, including reduced apoptosis and increased cell numbers. On day 7, more expanded cloned blastocysts were observed in the Vc-treated group. We speculated that Vc treatment might relax chromatin of donor cells by DNA or histone demethylation [13,14,36], and this was demonstrated by MNase digestion in this study. This change allowed the transcriptional factors to approach their targets more easily. However, the effect of Vc-treated donor cells on the full-term development of cloned bovine embryos has not been examined. Researches showed that Vc enhanced pre- and post-implantation development by treating cloned embryos in both *mouse* [37] and *pig* [33].

Considering the potential role of Vc in transcriptional activation, RNA sequencing was used to identify the genes that were upregulated in cloned embryos by Vc treatment. GO and KEGG analysis indicated that Vc treatment promoted the expression of genes coding for the cell–substrate adherens junction in the cloned embryos. The adherens junction is involved in cell recognition, adhesion, morphogenesis, and tissue integrity [38,39]. During early embryogenesis, the enhanced adherens junction by Vc treatment should be beneficial for communication between blastomeres and the formation of blastocysts.

Compared to oocytes, we found that autophagy was activated in the IVF two-cell embryos. However, the activation of autophagy was weaker in the cloned two-cell embryos than in the IVF two-cell embryos. As expected, we found that induced autophagy by rapamycin addition promoted the formation of cloned blastocysts. Surprisingly, the autophagy activation was rescued by Vc treatment in the cloned two-cell embryos. It has been shown that Vc accelerates the degradation of intra- and extracellular proteins targeted to the lysosomal lumen by autophagic and heterophagic pathways in human astrocyte glial cells [40]. It also rescues bone marrow stromal cells from oxidative stress by promoting the autophagic flux by *SVCT2* [41]. Considering the improved cloning efficiency in the *mouse* due to induced autophagy [42], we believed that the promoted autophagy activation by Vc treatment would benefit the cloned embryo development in *cattle*.

In this study, we selected the genes that were aberrantly expressed in the cloned embryos but restored by Vc-treated donor cells. The zinc-finger protein ZNF641 expression decreased in the cloned two-cell embryos and was rescued by Vc treatment. It is known that some zygotic genes are not activated in the cloned bovine embryos because of incomplete nuclear reprogramming [5,43,44]. To examine the benefit of *ZNF641* activation, it was ectopically overexpressed in early embryos. Although *ZNF641* compensation didn’t obviously promote the formation of cloned blastocysts, the quality of derived blastocysts was significantly improved. Thus, the restored *ZNF641* expression by Vc treatment should be beneficial for the development of the cloned embryo.

Interestingly, some aberrantly expressed lncRNAs were restored by Vc treatment in the cloned embryos. LncRNAs are involved in the regulation of gene transcription [45], post-transcription [46], and protein stability [47,48]. Moreover, lncRNAs can also regulate autophagy [49]. Thus, the abnormally expressed lncRNAs might impair the developmental potential of cloned embryos. Roles of the lncRNAs rescued by Vc treatment need to be further investigated in the cloned embryos.

It has been demonstrated that RNA-editing is necessary for the normal development of mammalian embryos [50,51,52]. RNA editing promotes developmental stage–specific gene and lncRNA expression by regulating silencing via RNA interference (RNAi) [53]. This study revealed that some deficient RNA-editing sites that occurred in the cloned two-cell embryos were improved by Vc treatment. However, Vc treatment didn’t change the transcript level of *ADAR* (a central gene responsible for RNA-editing [54]) in cloned embryos. Thus, it might be realized by enhancing *ADAR* enzyme activity or by other RNA-editing processes.

In conclusion, the present study revealed that Vc treatment can relax chromatin of donor cells and improve gene expression in the cloned embryos. Vc treatment promoted the expression of genes involved in the cell–substrate adherens junction and facilitated the activation of autophagy in cloned embryos. The expression of some coding genes and lncRNAs were restored by Vc treatment. It also rescued the RNA-editing of some deficient sites in the cloned embryos. Therefore, we demonstrated that Vc treatment has the potential to improve cloning efficiency at the transcriptional level. However, its ultimate benefit for full-term embryonic development remains to be investigated.

## Figures and Tables

**Table 1:** Vc treatment improved the preimplantation development of cloned embryos.

Groups	No. of Oocytes	Fertilized or Fused Embryos (%)	Cleavage (day 1) (%)	Cleavage (day 2) (%)	Blastocysts (%)
IVF	276	65.1 ± 2.9	68.5 ± 1.8	87.9 ± 2.0	47.8 ± 1.9
NTC	257	76.6 ± 2.7	51.8 ± 3.0*	75.2 ± 2.3*	27.6 ± 2.5*
NTT	292	81.7 ± 2.3	65.0 ± 2.5	80.3 ± 2.3	42.2 ± 2.0

Rates for cleavage and blastocyst formation were calculated relative to numbers of fertilized or fused embryos. In the same column, an asterisk superscript indicates difference compared to IVF (*p* < 0.05). IVF, in vitro fertilization; NTC, nuclear transfer embryos with control donor cells; NTT, nuclear transfer embryos with donor cells treated by vitamin C.

**Table 2:** Rapamycin addition promoted the formation of cloned blastocysts.

Groups	No. of Reconstructed Embryos	Rates of Blastocyst Formation (%)
Control	134	25.6 ± 1.7
Rapamycin	141	36.0 ± 2.1*

In the same column, an asterisk superscript indicates a difference (*p* < 0.05). Control: the cloned embryos treated by an equal volume of DMSO; rapamycin: the cloned embryos treated by rapamycin.

**Table 3:** Effects of ZNF641 overexpression on the development and quality of cloned embryos.

Groups	Rates of Blastocyst Formation (%)	Cell Numbers in a Blastocyst	Apoptotic Cell Numbers in a Blastocyst
IVF	48.2 ± 2.2	108.7 ± 3.9	2.4 ± 1.0
NTC	24.3 ± 2.6*	85.2 ± 3.7*	5.5 ± 1.2*
ZNF641	30.8 ± 3.0*	99.6 ± 3.2	3.1 ± 1.1

In the same column, an asterisk superscript indicates difference compared to IVF (*p* < 0.05). IVF: in vitro fertilized embryos; NTC: the cloned embryos; ZNF641: the cloned embryos injected with ZNF641 mRNA.

**Table 4:** Vc treatment rescued some deficient RNA-editing sites in cloned two-cell embryos.

#Chromosome	Position	Var_Location	Gene_Symbol
4	112989816	3_UTR	*PDIA4*
4	112989835	3_UTR	*PDIA4*
18	59002360	exon	*ENSBTAG00000011926*
26	47119212	intron	*DOCK1*
3	25225847	intron	*SPAG17*
8	10280038	intron	*PNOC*
6	16073325	intron	*ENPEP*
6	31334789	intergenic	*BMPR1B-PDLIM5*
14	10170314	intergenic	*ENSBTAG00000007736-ENSBTAG00000047834*
2	93412352	intergenic	*U6-ENSBTAG00000046521*
19	15089622	intergenic	*snoZ30-SLFN14*
7	100884170	intergenic	*CHD1-FAM174A*
18	25084111	intergenic	*HERPUD1-NLRC5*
7	5334946	intergenic	*FCHO1-MAP1S*

Var_location means the sequence feature where a variation is located.

## Supplementary Materials

Supplementary materials can be found at https://www.mdpi.com/1422-0067/20/11/2628/s1. Supplemental Information includes three figures and one table. Figure S1. The saturation of mapped reads for each sample. Figure S2. Distribution of mapped reads for each sample. Figure S3. Effects of Vc treatment on the gene expression in cloned embryos. Table S1. Primer sequences for real time PCR.

## Abbreviations

Vc	vitamin C
IVF	*in vitro* fertilization
SCNT	somatic cell nuclear transfer
NTC	nuclear transfer embryos with control donor cells
NTT	nuclear transfer embryos with donor cells treated by vitamin C
GO	gene ontology
KEGG	FPKM fragments per kilobase of exon per million reads mapped
FPKM	fragments per kilobase of exon per million reads mapped
lncRNA	long non-coding RNA
RT-qPCR	real-time quantitative PCR
FC	fold change
