# Supplementary material for: Transcriptome Analyses Reveal Effects of Vitamin C-Treated Donor Cells on Cloned Bovine Embryo Development
Source: Int J Mol Sci. 2019 May 28;20(11):2628. doi: 10.3390/ijms20112628 (PMC6600264; doi:10.3390/ijms20112628)
Supplement: Supplementary file 1 [file ijms-20-02628-s001.pdf]

Supplemental Figures

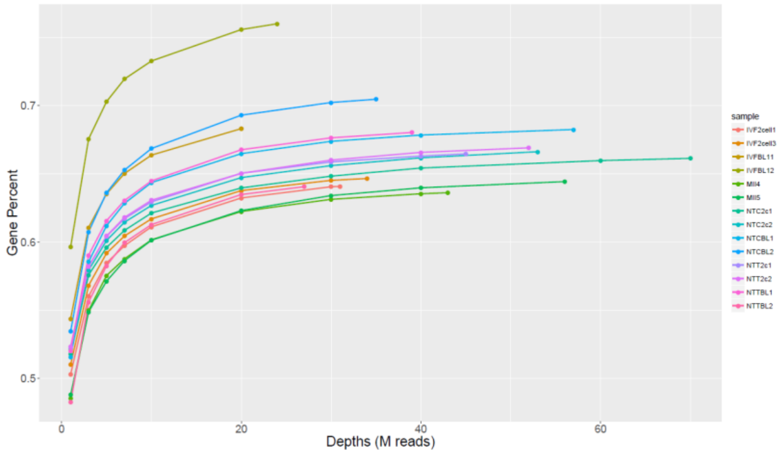

Figure. S1. The saturation of mapped reads for each sample.

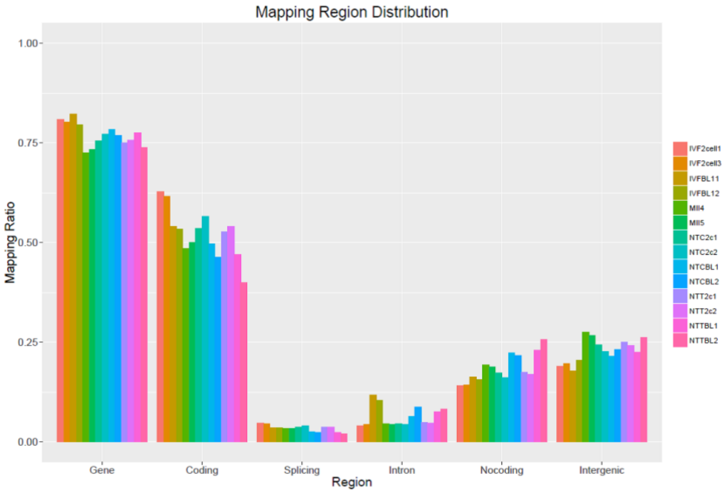

Figure. S2. Distribution of mapped reads for each sample.

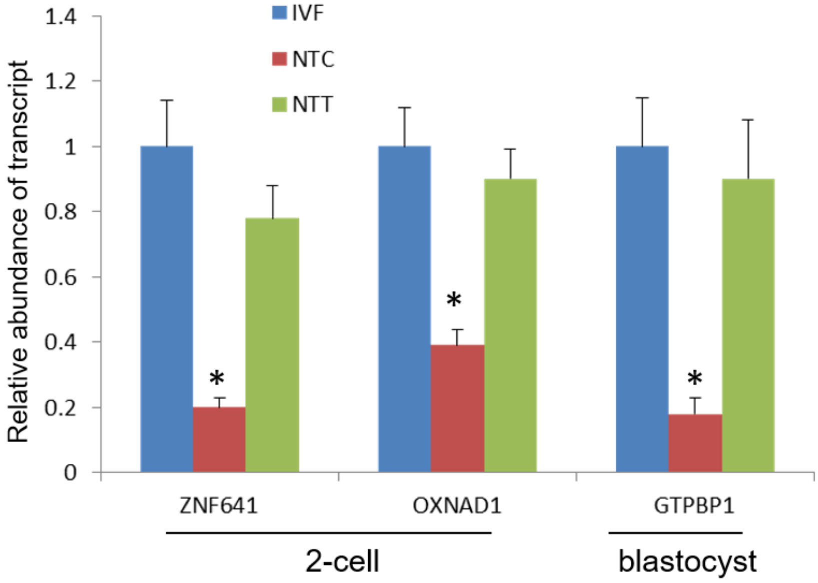

Figure S3. Effects of Vc treatment on the gene expression in cloned embryos. Bar graph illustrates the decreased expression of several genes in cloned embryos compared to their IVF counterparts; they were rescued in cloned embryos by Vc treatment. In the same cluster, an asterisk superscript indicates difference compared to the IVF counterpart ($P < 0.05$). NTC means non-treated cloned embryos and NTT means cloned embryo derived from Vc-treated donor cells.

Supplemental Table

Table S1. Primer sequences for real time PCR.

Genes	Primer sequences (5´- 3´)	Tann[a] (°C)
H2A	F[b]: GAGGAGCTGAACAAGCTGTTG	60
	R[c]: TTGTGGTGGCTCTCAGTCTTC	
SOX2	F: GGTTGACATCGTTGGTAATTTATAATAGC	60
	R: CACAGTAATTTCATGTTGGTTTTTCA	
POU5F1	F: CCACCCTGCAGCAAATTAGC	60
	R: CCACACTCGGACCACGTCTT	
NANOG	F: CGTGTCCTTGCAAACGTCAT	60
	R: CTGTCTCTCCTCTTCCCTCCTC	
CDX2	F: GCAAAGGAAAGGAAAATCAACAA	60
	R: GGGCTCTGGGACGCTTCT	
ZNF641	F: AGAAGGCTCAGTCCACTCCC	60
	R: AGGGAGACCACTATCCCACAG	
OXNAD1	F: TTCGCCAGGAGGTCGTTTC	60
	R: AGGAGGGTGGTTTGCATGTT	
GTPBP1	F: AGCCTGCCGAGTTTCAGATT	60
	R: CAGGCGTGGAGAAACCATCA	

[a] Annealing temperature.

[b] Forward primer.

[c] Reverse primer.
